# Supplementary material for: Bacillus thuringiensis Cry4Ba Insecticidal ToxinExploits Leu615 in Its C-Terminal Domain to Interact with a Target Receptor—Aedes aegypti Membrane-Bound Alkaline Phosphatase
Source: Toxins (Basel). 2021 Aug 9;13(8):553. doi: 10.3390/toxins13080553 (PMC8402544; doi:10.3390/toxins13080553)
Supplement: Supplementary file 1 [file toxins-13-00553-s001.zip › toxins-1266864 -SI.pdf]

Supplementary Materials: *Bacillus thuringiensis* Cry4Ba Insecticidal ToxinExploits Leu[615] in Its C-terminal Domain to Interact with a Target Receptor—*Aedes aegypti* Membrane-Bound Alkaline Phosphatase

Anon Thammasittirong, Sutticha Na-Ranong Thammasittirong, Chompounoot Imtong, Sathapat Charoenjotivadhanakul, Somsri Sakdee, Hui-Chun Li, Siriporn Okonogi and Chanan Angsuthanasombat

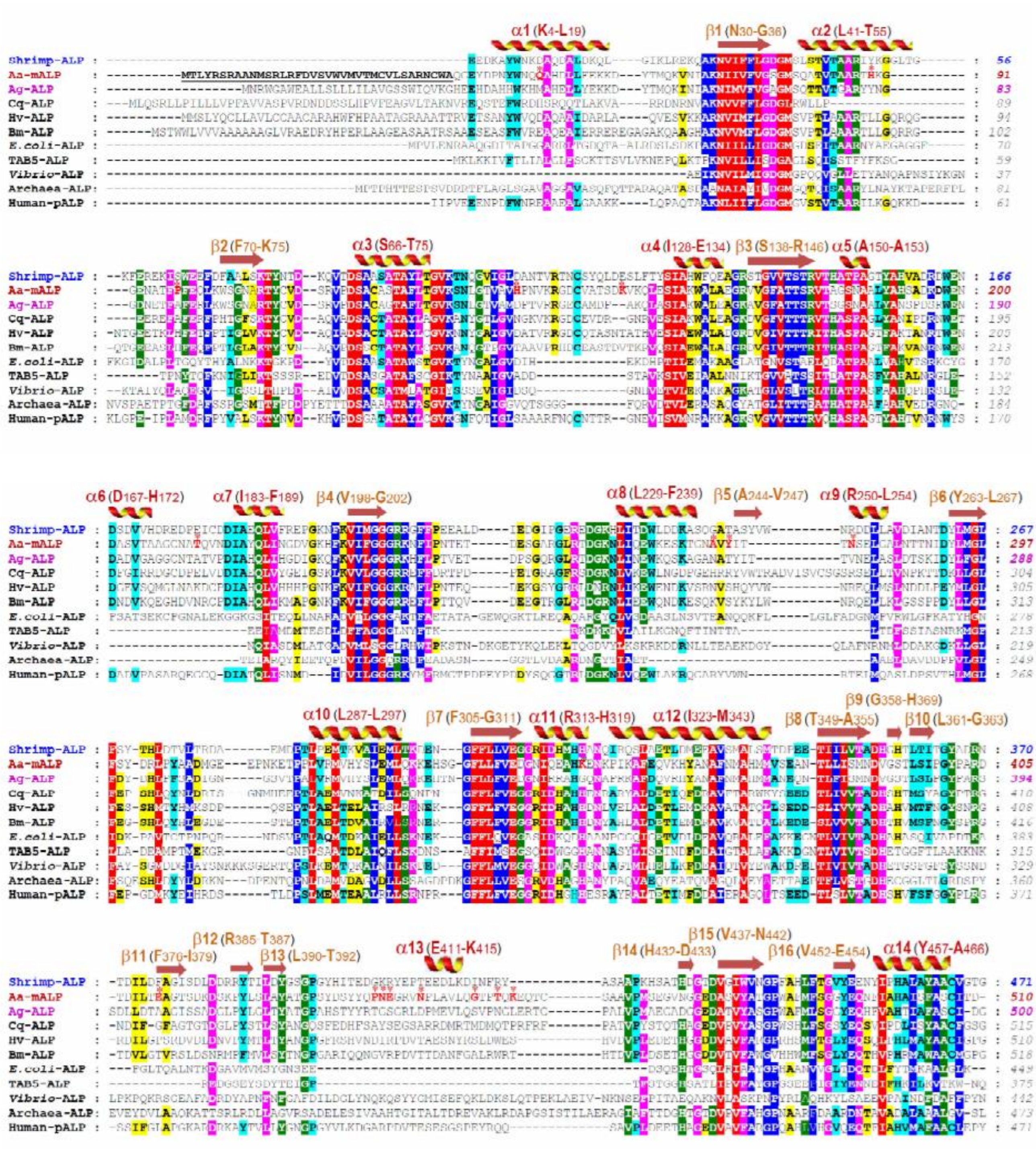

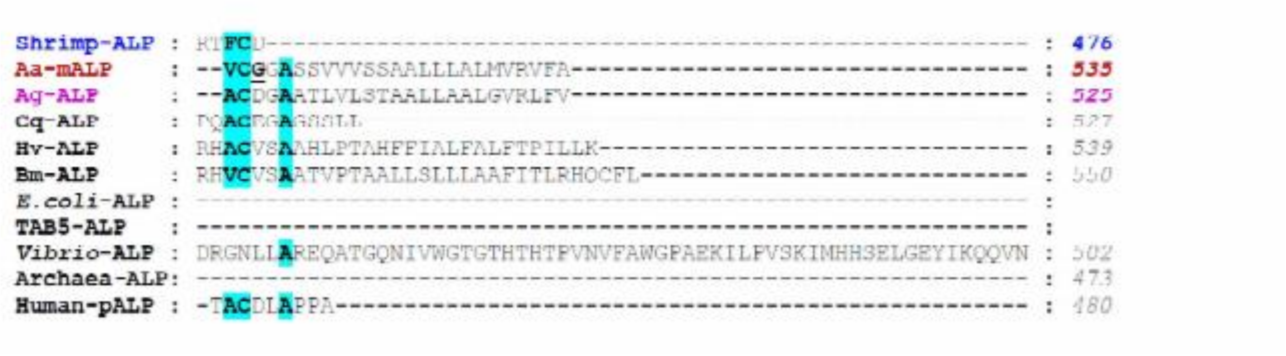

Figure S1. Multiple sequence alignments of Alps. The deduced amino acid sequence of Aa-mALP was compared with ALPs from other organisms including the sequences of known 3D-structures ALPs (shrimp-ALP, E. coli-ALP, TAB5-ALP, Vibrio-ALP, Archaea-ALP and human-pALP) and that of undetermined structures from other insect species (Ag-ALP, Cq-ALP, HvALP and Bm-ALP). Shrimp-ALP was used as a reference sequence for secondary structures which are shown on top of the corresponding sequence. The predicted signal sequence and GPI-anchor site of Aa-mALP are underlined. Amino acids are shaded red, blue, pink, green, cyan and yellow to donate degree of homology (11/11), (10/11), (9/11), (8/11), (7/11), (6/11), respectively.

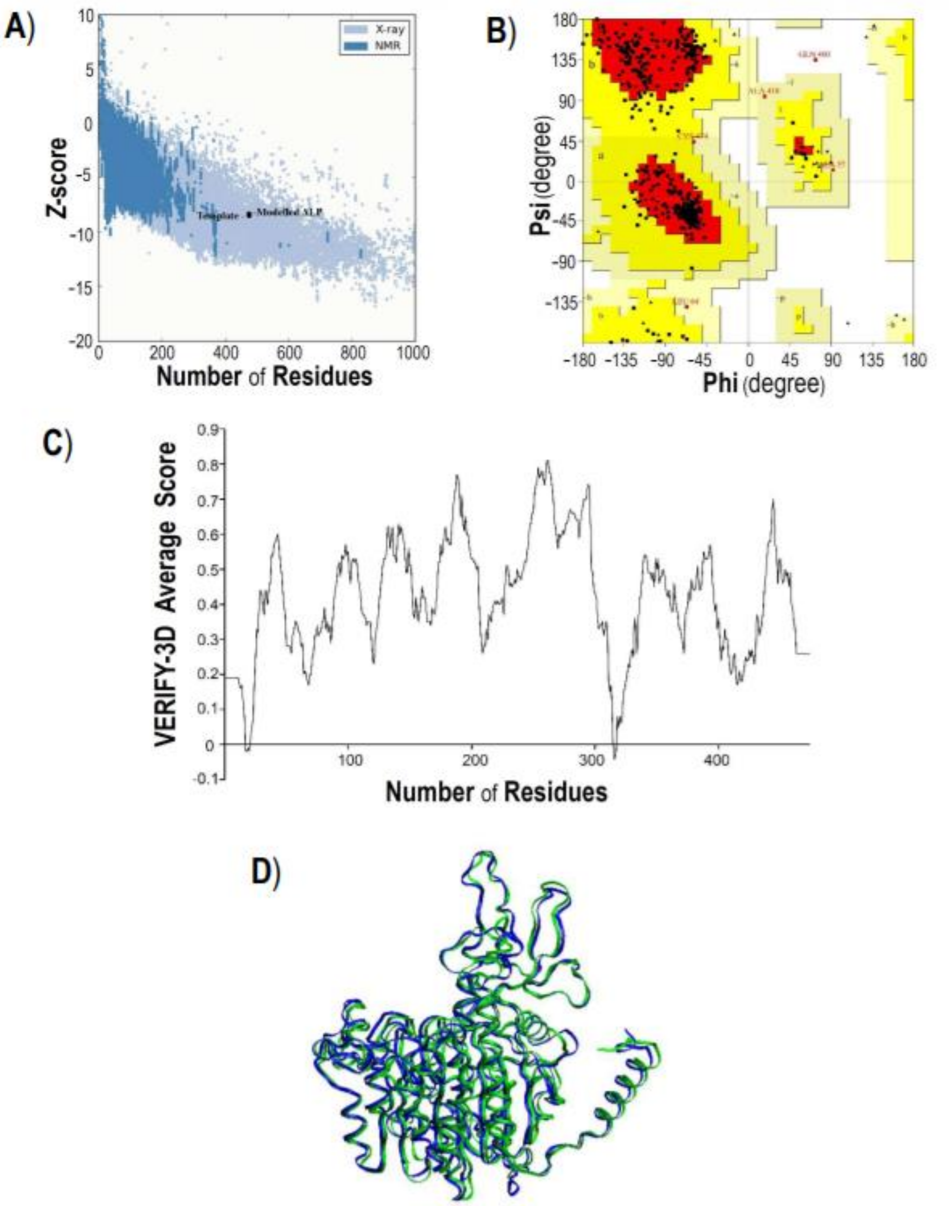

Figure S2. (A) The PROSA energy plot of the Aa-mALP model, indicating an overall quality score (Z-score) of the 3D model compared with that of all experimental determined structures (via X-ray and NMR). Z-score of the template (shrimp-ALP) was also determined and shown in the same plot. (B). The Ramachandran plot of the Aa-mALP model, in which 355 residues are in the most favored region (85.5%), 55 residues are in an additionally allowed region (13.3%), 4 residues are in a generously allowed region (1.0%) and 1 residues is in a disallowed region (0.2%). (C). VERIFY-3D validation of the Aa-mALP model, showing that 93.5% of residues have an average 3D–1D score ≥ 0.2. (D) Superposition of two Aa-mALP modeled 3D structures which were constructed based on single template (green) and multiple template (blue).

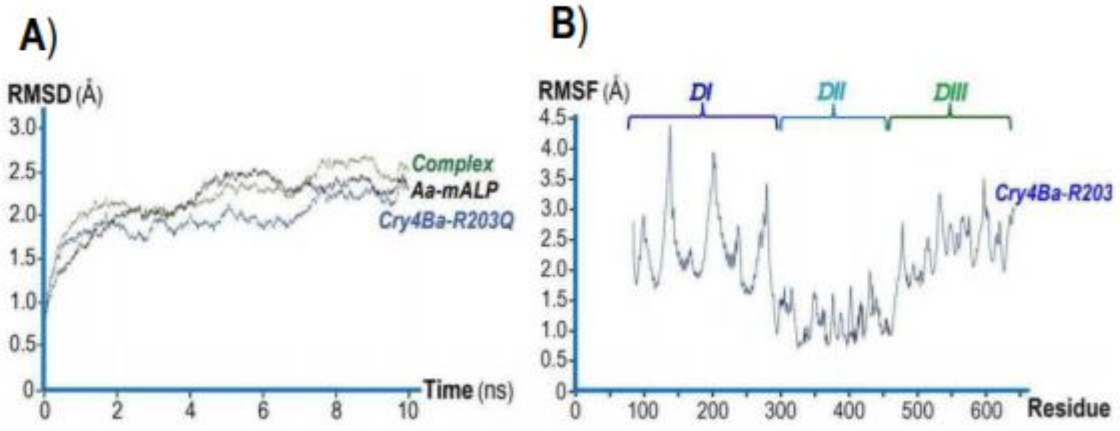

Figure S3. MD profile of the Cry4Ba-Aa-mALP docking complex, showing (A) all atoms RMSD in 10-ns MD simulations of the docking complex (green line), Cry4Ba-R203Q (blue line) and Aa-mALP (black line) together with (B) fluctuation (RMSF) of Cry4Ba-R203Q during the MD simulations.

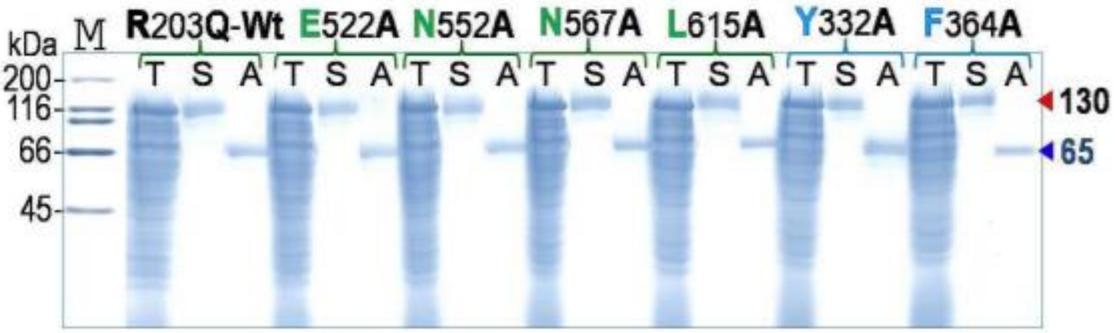

Figure S4. SDS-PAGE (Coomassie brilliant blue-stained 12% gel) analysis of the Cry4Ba-R203Q toxin and its corresponding mutant toxins as indicated. T, Total crude cell lysate containing the 130-kDa over-expressed protoxin; S, the 130-kDa solubilized protoxin; A, the 65-kDa trypsin-activated toxin. M, molecular mass standards.

Table S1. Complementary pairs of mutagenic primers for Alasubstitutions of residues in Cry4Ba-DII and III.

Domain site	Primer[a]	Sequence[b]	Restriction
II	Y332A-*f*	5'-CAATACTATA**GCGC**AAGATTTAAG-3'	*Hha*I
	Y332A-*r*	5'-CTTAAATCTT**GCGC**TATAGTATTG-3'	
	F364A-*f*	5'-GAAGTTCTGGT**GCC**GGTTCAAATCTTAC-3'	*Hpa*II
	F364A-*r*	5'-GATTTGAACC**GGC**ACCAGAACTTCC-3'	
III	E522A-*f*	5'-AGAATGG**CC**ATTCAATGTAAAACAA-3'	*Hae*I
	E522A-*r*	5'-CATTGAAT**GG**CCATTCTGCCTGA-3'	
	N552A-*f*	5'-TGTATTG**GC**TGTATC**T**TATGTATTACAAGGA-3'	*Nde*I
	N552A-*r*	5'-AATACATA**A**GATACA**GC**CAATACAATTGGAC-3'	
	N576A-*f*	5'-CGTTTTC**T**AGACCT**GC**TAATATAATACCTACA-3'	*Xba*I
	N576A-*r*	5'-TATTATATTA**GC**AGGTCT**A**GAAAACGTAGATTC-3'	
	L615A-*f*	5'-CAACCA**GC**AAACATGACTTC**G**AATAATCAAGTG-3'	*Bst*BI
	L615A-*r*	5'-TGATTAT**TC**GAAGTCATGTTT**GC**TGGTTGAATAG-3'	

[a] f and r represent forward and reversed primers, respectively. [b] Underlined and double underlined bases represent the recognition sites introduced and eliminated for restriction analysis, respectively.
